# Computer-Aided Strategies for Determining the Amino Acid Composition of Medium for Chinese Hamster Ovary Cell-Based Biomanufacturing Platforms

**DOI:** 10.3390/ijms20215464

**Published:** 2019-11-02

**Authors:** Bergthor Traustason, Matthew Cheeks, Duygu Dikicioglu

**Affiliations:** 1 Department of Chemical Engineering and Biotechnology, University of Cambridge, Cambridge CB3 0AS, UK; bt362@cam.ac.uk; 2 Cell Sciences, Biopharmaceutical Development, AstraZeneca, Cambridge CB21 6GH, UK; cheeksm@medimmune.com

**Keywords:** Chinese hamster ovary, medium development, amino acid, heterologous expression, biologics, biomanufacturing, metabolic models, design of experiments

## Abstract

Chinese hamster ovary (CHO) cells are used for the production of the majority of biopharmaceutical drugs, and thus have remained the standard industry host for the past three decades. The amino acid composition of the medium plays a key role in commercial scale biologics manufacturing, as amino acids constitute the building blocks of both endogenous and heterologous proteins, are involved in metabolic and non-metabolic pathways, and can act as main sources of nitrogen and carbon under certain conditions. As biomanufactured proteins become increasingly complex, the adoption of model-based approaches become ever more popular in complementing the challenging task of medium development. The extensively studied amino acid metabolism is exceptionally suitable for such model-driven analyses, and although still limited in practice, the development of these strategies is gaining attention, particularly in this domain. This paper provides a review of recent efforts. We first provide an overview of the widely adopted practice, and move on to describe the model-driven approaches employed for the improvement and optimization of the external amino acid supply in light of cellular amino acid demand. We conclude by proposing the likely prevalent direction the field is heading towards, providing a critical evaluation of the current state and the future challenges and considerations.

## 1. Introduction

The global biopharmaceutical market is growing rapidly in light of recent advances in the field leading to the widespread utilization of biopharmaceuticals in the treatment of numerous diseases [1]. The majority of biopharmaceutical drugs are currently produced using Chinese hamster ovary (CHO) cells, which have remained the standard industry host for the past three decades. CHO cells are predominantly used as expression hosts for recombinant monoclonal antibody (mAb) production, which comprises the fastest growing segment of the biopharmaceutical industry [2].

CHO cells require suitable growth conditions in order to produce and secrete a required amount of the desired recombinant protein. The nutrient components provided in the culture medium comprise one of the most important factors in establishing an optimal growth environment for the cells, and therefore this task constitutes a central activity in the design of upstream cell culturing of biopharmaceutical processes [3]. Among these components, amino acids constitute the building blocks of both the recombinant protein and of the native CHO proteins, which make up ca. 70% of the dry cell mass [3,4]. Mammalian cells can only produce some of the amino acids that are required for protein synthesis, while others, the essential amino acids, need to be supplemented externally. Amino acids act as precursors for a multitude of intermediates in a large number of metabolic pathways, and as important sources of nitrogen and potentially carbon when other sources are limited. It is, therefore, imperative that the CHO cells are supplemented with the sufficient amount of essential amino acids to sustain continued survival, growth, and proliferation, and with non-essential amino acids to facilitate the favorable use of resources. The extracellular provision of non-essential amino acids limits the extent of energy loss in amino acid biosynthetic pathways, and renders additional reducing power available for other biological processes in the cell, reducing additional metabolic burden on the CHO cell metabolism via the extensive use of biosynthetic amino acid routes. This, in turn, avoids potential constraints on the growth yield and cellular productivity.

In line with their relevance, amino acid metabolism and related metabolic and non-metabolic pathways of CHO cells have been extensively studied [5,6,7]. Consequently, the key significance of amino acids in medium design has been recognized for a long time, although, even after decades of industrial practice, there is still a huge potential for improvement of the growth environment for CHO cells [8]. No systematic procedure exists for the optimization of the CHO cell culture growth and production medium; several strategies followed by the industry led to the emergence of a vast number of different medium formulations, including those of amino acids, predominantly developed based on relevant experience. Comprehensive and insightful reviews are available on the subject [9].

**Figure 1:**
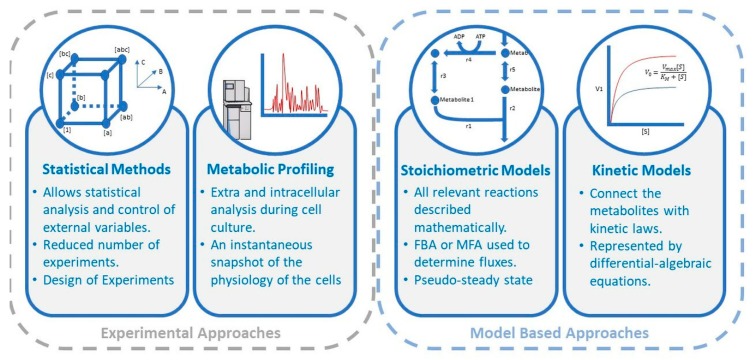
Overview of the mainstream approaches currently used in the optimization of amino acid composition in culture medium for recombinant protein-producing Chinese hamster ovary (CHO) cells. Note that the approaches summarized above are those specifically relevant within the domain of tailoring medium composition for optimal utilization of amino acids within the domain of recombinant protein production by CHO cells. A plethora of alternative systematic strategies not limited to those covered here (beyond the scope of this Review) exist, which deal with different aspects of improving biomanufacturing platforms for CHO cell-based technologies. FBA: flux balance analysis; MFA: metabolic flux analysis; ADP: adenosine diphosphate; ATP: adenosine triphosphate; v: rate of reaction; [S]: substrate concentration; v_max_: maximum rate of reaction; K_M_: saturation constant.

Due to their economic importance, CHO cell-based biomanufacturing platforms have been the subject of extensive investigation for process optimization. Model-based strategies have been successfully adopted for improving upstream [10,11] and downstream [12,13] processing and conducting an economic evaluation of different strategies [14,15]. Excellent reviews exist on the subject [16,17]. This paper describes the latest and imminent efforts on the utilization of model-driven approaches in medium development, which provide mechanistic insight into the CHO production system and replace heuristic efforts with limited systematic abilities (Figure 1). Amino acids so far served as excellent test cases for such attempts, in light of the extensive knowledge on the amino acid metabolism, which allowed the construction of mechanistic models to draw conclusions from and translate them into process requirements. Consequently, the investigation of amino acid routes in conjunction with mechanistic models of the cell have been successfully integrated in model organisms such as the Baker’s yeast [18], and in industrial yeasts employed in recombinant protein production [19], although such efforts are fairly recent, and limited in number for industrially relevant mammalian cells, including CHO systems. Therefore, many resources focus on these efforts to improve the formulation of chemically defined media collectively within the domain of recombinant protein production, providing an overall view on a wide subject [20]. This work specifically aims to address these recent advances in the field, and focuses on the range of model-based applications and approaches assisting empirical efforts in the exclusive identification of amino acid requirements of CHO cells and consequent tailoring of their growth environment.

## 2. Brief Overview of the Established Practice in Determining the Amino Acid Composition of the CHO Cell Medium

Reports on improving the culture medium composition for CHO cells, specifically in relation to the amino acids, are relatively limited in number. This paucity of reports is strikingly unusual especially considering the widespread use and vast economic importance of CHO cells [3].

### 2.1. Statistical Methods: Utilizing Data-Driven Models in Experimental Design

Statistical design, also known as design-of-experiments, is arguably the most commonly employed strategy for medium development. Unfortunately most such studies for CHO cells focus on components such as those relevant for the carbon and energy metabolism [21,22,23,24] with only a limited number of studies specifically addressing amino acids. In line with this observation for statistical methodologies, search heuristics such as genetic algorithms commonly employed in medium development and optimization in various organisms [25] have not been utilized for improving CHO cell medium compositions, even the chemically defined cell-free formulations.

Low glutamine and high essential amino acid concentrations led to high mAb production against Botulinum A by CHO cells. An amino acid other than glutamine (analytically uncharacterized) was identified as the limiting component in a study that employed response surface methodology to evaluate medium-associated effects on viable cell density (as a proxy for mAb concentration) during coupled production and growth [26].

González et al. used the Plackett-Burman design method to optimize the contribution of ten amino acids to the rate of growth and monoclonal antibody production by Chinese hamster ovary cells. These 10 amino acids were specifically identified as per their superior uptake and utilization by the CHO cell metabolism [27]. The analysis was carried out both during exponential phase of growth through monitoring how the specific growth rate and the concentration of the viable cell concentration evolved over time, and during the phase where the cell population reached a very high density, through monitoring of the final monoclonal antibody product concentration and the specific productivity of the cell population. This investigation aimed to determine which among the ten amino acids were critical for supporting health growth of the population and product formation. In return, this information could be utilized for designing an amino acid supplementation regime, through which a superior culture could be achieved through medium improvements. The authors proposed a two-stage strategy for providing the cells with the key amino acids. One stage focused on achieving a healthy cell culture through focusing on growth optimization, and a second stage which optimizes product formation once a large cell population has been achieved.

More recently, Torkashvand et al. used the Plackett-Burman design to screen the effects of amino acids on promoting growth, and then optimized the medium concentrations of a combination of amino acids using response surface methodology to determine the feed composition [28].

Although statistics were employed in these studies, it would be very difficult to extract learning as to the nature of the potential benefits of employing different design approaches since these investigations focused on the empirical aspects of the study rather than providing an in-depth evaluation of the statistical tools employed. Consequently, they fail to compare and contrast different methods and provide reasoning for the selection of certain models over others, or point towards the utilization of favorable statistical designs, which could prove invaluable to attain systematic medium development strategies.

### 2.2. Metabolic Profiling

Another method widely used in the industry to understand the impact of amino acid composition on mAb production is exo- and endo-metabolic profiling. Such methods are purely empirical and are mainly utilized to improve an established platform process, by suggesting specific nutrients to be added during the course of a cultivation, for example, rather than to conduct a broad de novo exploration.

A study on the identification and elimination of cellular and process bottlenecks that prevent high levels of antibody production by CHO cells employed metabolic profiling to identify the depletion of tyrosine, a key amino acid for protein synthesis, as the bottleneck for production, which could successfully be alleviated by its additional supplementation in the feed [29]. A CHO cell culture supplied with glutamine and glucose was shown via metabolic profiling to deplete aspartate, cysteine, methionine, tryptophan, and tyrosine during antibody production. This analysis was used to implement a medium supplementation strategy doubling the amount of the depleted factors, and the culture performance was shown to improve without affecting the quality of the mAbs [30]. Unlike earlier studies, Sun et al. adopted metabolic profiling of amino acids as a top-down approach to develop an optimal medium formulation in response to culture performance [31]. Metabolic profiling was used not only to develop or fine-tune medium formulations, but also to identify bottlenecks leading to non-metabolic cellular processes. An analysis of the temporal amino acid concentration profiles of naive and recombinant CHO cell cultures showed the highest depletion rates for those amino acids that constitute the highest mass fractions of the mAb, and unexpectedly, also for alanine, and this was attributed to its importance for the metabolic processes related to recombinant protein synthesis. Significantly different amino acid demands were observed during the growth phase and the production phase for the CHO cell culture. However, these differences were not accompanied by statistically significant differences in rates of consumption [3].

### 2.3. Modification of the Amino Acid Transporters

The study of the role of cell membrane amino acid transporters (AATs) in CHO cell medium development has only recently gained attention. AATs mediate the cellular uptake of amino acids and take part in connecting compartmentalized metabolic pathways, energy generation and redox regulation [32]. Due to that reason, there are considerable opportunities in genetically engineering the activity of AATs in conjunction with the amino acid composition of the medium to improve mAb production by recombinant CHO cells. However, necessary information regarding the activity of the functional classes of AATs is still lacking, which renders current efforts exploratory rather than applied [33]. Our current understanding on the amino acid transporters or transporter families leads to the thought that they are highly specific with regards to their transport mechanism, substrate specificity, and even at times, to cell type/tissue specificity and the roles they play in different physiological functions [32]. Unfortunately, this information has not yet been utilized in tailoring an optimal medium or feeding strategy for CHO cells producing recombinant proteins in either empirical or model-based studies. The translation of such molecular insight into biotechnological understanding would nevertheless be essential in developing more elegant and systematic strategies for this long-standing quest.

The gene expression profiles of AAT-encoding genes in naive and mAb-producing CHO cells were investigated by RNA-seq analysis. The functional analyses via the use of transporter-specific chemical inhibitors of these transporters, whose gene expression profiles were obtained, showed that half of the 16 ATTs overexpressed in CHO cell populations were specifically upregulated in cells that were engineered for recombinant mAb production. The analyses showed that the cells were supplemented with the amino acids, which dominate the amino acid composition of the CHO cells that express the recombinant monoclonal antibody, through the use of their cognate amino acid transporters. This allowed the cells to avoid shortage of essential amino acids and to ensure constant activity of synthesizing proteins. Furthermore, it enabled the provision of amino acids as substrates for facilitating the coordinated antiport activity across transport channels [33,34]. However, these analyses were limited to the investigation of the expression levels of AAT-encoding genes. Although the activation or the suppression of relevant transduction pathways such as the mammalian Target of Rapamycin (mTOR) pathway, which plays an essential role in protein synthesis, has extensively been studied in mammalian systems [35], neither this notion, nor coupling this information with metabolome and transcriptome-based analysis of amino acid transport pathways, has yet been utilized in the context of optimizing medium requirements for recombinant protein production. Furthermore, the differences in the relative contribution of different amino acids and their cell-specific roles have not been explored in this context either.

## 3. Mechanistic Insight by Model-Assisted CHO Cell Medium Development and Optimization

Empirical methods for process improvement are often resource-intensive [36]. Furthermore, a recombinant protein that is inherently complex, or one that imposes stringent requirements on the host metabolism necessitates a rational approach to medium tailored for the mammalian host [37]. Consequently, in light of the increasing demand and the expanding market for evermore capable and complex biologics products, there is emerging interest in using model-based approaches to assist and guide experimentation to achieve process improvement in terms of both productivity and product quality. As in process development, models facilitate hypothesis generation and preliminary testing of sprouting ideas, as well as the development and discovery of novel methods in medium development. However, despite their broad popularity in CHO cell culture development and production, the utilization of mechanistic models in medium development and optimization was rather limited, and even more so within the domain of the investigation of the complete amino acid profile [38,39].

### 3.1. Kinetic Models to Assist CHO Cell Upstream Bioprocess Development and Control

Understanding the dynamic behavior of the system is extremely useful in bioprocess design and improvement, and kinetic models are extremely valuable for this purpose since they establish relationships between metabolites through kinetic laws, which are represented by differential algebraic equations. Kinetic models of biological systems are often complex, and there is a paucity of rate constants describing kinetic relationships across all scales of life. Therefore, accurate kinetic models are usually available for only extremely well-studied metabolic pathways, generally those pertaining to the central carbon metabolism [40]. 

On the other hand, simple Monod-type kinetics are often employed to describe the cellular uptake of main macronutrients and the rate of growth and the secretion of main metabolic byproducts, and CHO cells are no exception. Lopez-Meza et al. studied the kinetics of cell growth, consumption of glucose, mAb product formation and lactate secretion in naive and recombinant CHO cells [41]. Monod-type growth kinetics were utilized and a variant of the Luedeking-Piret model was proposed to associate the rate of product formation to growth rate and the cell density. The results showed that simple kinetic models could successfully explain cellular growth, product formation, and glucose consumption. A modified Monod-type model proposed for cellular growth was able to account for a threshold glucose concentration of 0.6 g/L below which no growth was observed.

Monod-type models on bioprocesses, which specifically focus on the reaction kinetics of the mammalian amino acid metabolism, are available, but these studies are very limited in number as well as in context [42,43,44]. Selisteanu et al. used a dynamic model to describe mAb production in CHO cells. They estimated the kinetic parameters for a set of reactions that utilize alanine, glutamine, glutamate, aspartate, asparagine, and proline by minimizing an error function with a particle swarm optimization algorithm. The statistical analysis they performed demonstrated that the proposed estimation method was robust against normally distributed noisy measurements, and that it provided acceptably accurate results. [36,45]. A kinetic model of the central metabolism of CHO cells comprised of 34 reactions was employed to investigate the role of amino acids in both the central carbon metabolism and the tricarboxylic acid (TCA) cycle, as well as on recombinant mAb production and cellular growth. The model was thus proposed as an in silico platform, which could be challenged for optimizing medium composition and fed-batch culture strategies, although the authors did not attempt to make such projections in their analysis [46].

Simon and Karim used the extended Kalman filter on kinetic equations for CHO cell culture to estimate the number (or the density) of viable cells and the concentrations of glutamine and asparagine. These estimated variables were then fed to a neural network that approximated the concentration of apoptotic cells in a bioreactor. A controller could then be activated when the apoptotic cell concentration was predicted to reach a certain value [47].

Understanding the dynamic behavior of the system is extremely useful in bioprocess design and improvement, and thus rendering kinetic models valuable in the exploration of upstream processing (Table 1). However, the disadvantage of kinetic models is that non-linear equations can render the solution process difficult and computationally expensive, and that it may become very difficult to recreate exactly the dynamic interactions among the various components in an intracellular environment [48].

### 3.2. Stoichiometry-Based Modelling to Explore Amino Acid Requirements for Medium Development and Bioprocess Design

Stoichiometric models provide a different type of approach to the problem at hand through mathematically describing all relevant reactions in a system of interest. The material flow rate (i.e., the flux) through each of these reactions is then determined by methods such as metabolic flux analysis (MFA) and flux balance analysis (FBA). Both FBA and MFA constrain the network of reactions to respect the laws of conservation of mass and energy, as well as the observed conditions in the studied environment. They rely on the assumption of pseudo-steady state, which states that the accumulation of intracellular metabolites would be negligible compared to the observed fluxes [49,50]. MFA is employed to analyze networks that are fully defined by measured extracellular reactions, for example by isotopic-labeling. On the other hand, FBA methodology, coupled with linear programming, was developed specifically for the analysis of underdetermined systems, and thus are suitable for the analysis of genome-scale reconstructions of the metabolism.

MFA was utilized to some extent also for optimizing the concentration of non-amino acid medium components [51,52,53,54]. The adoption of similar techniques for amino acids is rather limited. Most work carried out in this domain focused on the supplementation of a single amino acid, namely glutamine, primarily due to its role in the formation of the unwanted byproduct, ammonia [55,56,57,58]. 

Xing et al. used MFA to modify the amino acid composition of the medium for a mAb-producing CHO cell culture. MFA was performed using 32 specific metabolic rates derived from two semi-steady states of continuous culture. It was suggested to modify the formulation by reducing the concentrations of arginine, alanine, glutamine, and glycine and increasing the concentrations of methionine, tryptophan, asparagine, and serine. The peak cell density and the product concentration were shown to improve by 55% and 27%, respectively, in cultures grown in the modified medium [59].

Recently, elementary flux mode analysis coupled with kinetic modelling was employed on a 126-reaction network of the CHO cell central metabolism. The metabolic response was tested against different scenarios that introduce variations in amino acid availability using this poly-pathway model [60].

Selvarasu et al. combined metabolomics and genome-scale modeling in an approach to gain new insight into the molecular mechanisms that occur within Chinese hamster ovary cell cultures that were grown in fed-batch mode. Former studies on the intracellular and extracellular metabolite profiling of these cells were employed to generate a list of metabolites, which could be strongly associated with limitations on the growth of a cell culture. A derivative of the mouse genome-scale metabolic network model was generated to investigate the key metabolic functions of CHO cells, and this new model was fine-tuned by incorporating cell-specific information that arose from CHO cDNA annotation collected by the research group. The analysis on the biomass composition revealed that the amino acid content of CHO cells was different from that of other mammalian cells. The subsequent in silico modeling characterized the intracellular metabolic behavior, and allowed further exploration of the pathways that are relevant for understanding growth limitations and identifying factors that were potentially growth-limiting [4].

Following up on these initial efforts, Hefzi et al. reconstructed the metabolic pathways in CHO and associated them with more than 1700 genes in its genome [61]. This genome-scale metabolic model of the CHO cell community comprised of 6663 metabolic reactions involving 2341 unique metabolites provides a systematic representation of the biochemical basis for growth and recombinant protein production in CHO cells. The mechanistic link between metabolic reactions and the enzymes catalyzing those reactions allows for the effective integration of orthogonal data types, such as metabolomics, genetic variants, transcriptomics, and phenotypic information. Following up on this development, an important progress in utilization of genome-scale metabolic models for medium optimization and development has recently been published. The community model of the CHO cell metabolism was curated extensively, and was adapted to conform to the metabolic profiles of high-yielding CHO cell lines used in industrial protein production. Constraining this modified model with the transport rates of twenty four metabolites that were measured on daily basis in four independent bench scale fed-batch cell culture processes for mAb production was shown to predict the fluxes calculated from exo-metabolomic data and the growth rate with reasonable accuracy. These predictions allowed the identification of potential routes towards improving the medium composition; one such suggestion was to reduce the amount of asparagine supplementation in the medium [62].

In another recent study, Fouladiha et al. employed this model and developed a method, which proposed different feeding strategies to improve monoclonal antibody production by CHO cells as hosts for a total of seven amino acids (GLU, ASP, LYS, TRP, THR, VAL, and HIS). Their methodology was focused on conducting a form of scanning of flux variabilities based on the use of an enforced objective flux [63]. This was a pioneering study in the sense that the genome-scale metabolic model of the Chinese Hamster Ovary cells was used for the first time with the specific purpose of designing a feeding strategy, and threonine was suggested as an important metabolic constraint, which could significantly improve mAb production in CHO cells. 

Another recent in silico investigation of the amino acid requirements of CHO cells during the expression of a range of antibody products through the use of genome-scale metabolic modelling by Traustason showed that the extracellular provision of non-essential amino acids, particularly that of cysteine and tyrosine, had a positive effect on CHO cell growth. This extracellular supplementation was thought to reduce the extent of energy loss in the amino acid biosynthetic pathways, thus rendering additional reducing power and energy available for other biological processes within the cell, leading to growth and growth-coupled antibody production. An analysis of a range of antibody production systems (alemtuzumab, trastuzumab, blinatumomab, ibritumomab tiuxetan, and denileukin diftitox) indicated that size alone did not explain the differences in the rates of secretion of different products, and that the amino acid content of the product also had a substantial contribution. Furthermore, the amino acid requirements of the medium did not match with the amino acid content of the antibody product indicating that blindly increasing the composition of the amino acids with the highest composition in the target protein was not necessarily the best strategy. This indicated that optimization of the amino acid formulation of the medium is a multi-parametric problem, which necessitates taking metabolic complexity into account, in order to narrow down the search space for experimental analysis [64].

Although a combination of machine learning techniques and metabolic modeling was recently employed to estimate lactate production in CHO cell cultures [65], these attempts, have not yet been extended to the domain of amino acid-related pathways, and consequently in the design of their medium in light of such metabolic requirements identified by these complex predictions. These efforts are summarized in Table 2.

## 4. Discussion

This paper provides an overview of the existing literature on our current understanding of improving recombinant protein production by CHO cells through the use of various digital platforms and model-driven regimes. The most striking feature encountered by this investigation is the limited availability of such studies in contrast to a plethora of empirical analyses available in the literature. This implies that the utilization of computer-aided model-based strategies in this field is still at its infancy, granting the field has gained particular momentum within the recent years. Most of the above-mentioned studies rely on the use of empirical techniques in conjunction with rational strategies to generate an outcome. However, they fall short in providing an in-depth critical evaluation of the adopted techniques or approaches, which could facilitate the development of a structured and systematic methodology to serve as a preliminary guideline for future studies that would focus on similar type of problems. This shortfall imposes a challenge for the scientific progress in the field as well as for the industrial development of novel biologics products, since both research and industry operate on tight and resource-constrained schedules. Moreover, we discern an immediate need for studies that specifically focus on evaluating and conducting a critical comparison of and benchmarking these computational methodologies to assist the end-users of these technologies in exploring their options and during their decision-making process.

Huge efforts have been made to bridge the evident gap between model-based approaches and empirical methods that are predominantly data-intensive in addressing different challenges faced in biomanufacturing platforms, let it be metabolic challenges [66,67,68] or the thermodynamic and kinetic limitations of the host system [69,70,71], or challenges associated with the biomanufacturing process itself [72,73,74,75,76]. Models can provide a mechanistic understanding of the processes, and suggest guidelines for narrowing down the search space for experimental analysis. Furthermore, they can assist the construction of a transient design space, which can expand, shrink, or shift, leading the way towards dynamic and adaptable product development in Quality-by-Design. High-throughput omics technologies coupled with the use of formal models can provide further insight into how changes to process inputs such as medium components would affect the output observations such as growth rate and productivity. System-based approaches and machine learning techniques are likely to create a paradigm shift in our understanding and handling of CHO cells within the domain of biopharmaceutical production, as they become widely used tools. Such hybrid approaches are expected to have superior ability to cope with the uncertainties associated with the stochasticity of the bioprocess as well as of the cell line development pipelines. In particular, the adoption of data-driven methodologies in conjunction with genome-scale metabolic modelling is anticipated to become an increasingly prevailing direction that the field will move towards [77].

Current practice in biomanufacturing of recombinant proteins inherently couples cellular growth and proliferation with recombinant protein production. This is a different approach to that of other practices that employ metabolic engineering for small molecule biomanufacturing where the cellular growth and product formation mechanisms would compete for available resources. In the event that a recombinant protein production process necessitates such decoupling, adoption of systematic model-based strategies could allow the fastest route towards the identification of the readjustments that need to be made to modify the existing processes by allowing a superior evaluation of the cellular performance the cell, and shorten the adaptation period.

In this domain, amino acids present a very suitable but also a challenging case. Understanding the role of the amino acid metabolism, and how this would translate into the amino acid requirements of CHO systems through model-based analysis, is certainly far more complex than acquiring a mechanistic understanding of how only key small molecules of a cell culture (glucose, lactate, glutamine, glutamate, and ammonium) evolve by modelling. Concurrently, the system is sufficiently well-studied to allow researchers dwell into this challenge.

The cellular requirements, of which the demand for different amino acids constitutes an important subset, can vary substantially for the production of different biopharmaceuticals, rendering the use of systematic and model-based approaches of prime interest to handle the cellular burden associated with the production of different types of proteins. The adoption and standardization of these recent technologies and advances will, without doubt, improve our understanding of the cellular refunctioning of the amino acids and assist biopharmaceutical process development in light of these findings.

## Figures and Tables

**Table 1:** Summary of the available kinetic model-based approaches employed in recombinant protein-producing CHO cell medium development and optimization.

Details on Kinetic Model	Metabolites/Pathways Involved	Reference
Monod-kinetics, Luedeking-Piret model for associating rates of growth and product formation	Growth, glucose uptake, lactate secretion, product formation	[41]
Dynamic model, particle-swarm optimization for parameter estimation	Alanine, glutamine, glutamate, aspartate, asparagine, and proline utilization, product formation	[36,45]
34-reaction model, multiplicative Michaelis-Menten kinetics	Role of amino acids in central carbon metabolism, tricarboxylic acid (TCA) cycle, recombinant monoclonal antibody (mAb) production, and cellular growth	[46]
Kalman filters for approximating the state variables represented by dynamic mathematical models, model predictive control on the apoptotic cell density, neural networks to express apoptotic cells as a function of state variables	Viable cell density, glutamine and asparagine concentration to predict apoptotic cell population	[47]

**Table 2:** Summary of the available stoichiometry-based models employed in recombinant protein-producing CHO cell medium development and optimization.

Method	Scale	Reference
Metabolic flux analysis + ^13^C analysis	Small scale: 272 reactions and 228 metabolites	[51]
Metabolic flux analysis + ^13^C analysis	Small scale: 58 reactions and 50 metabolites	[52]
Elementary flux mode analysis/extreme pathways	Small scale: 24 extracellular, 13 intracellular species, 35 reactions	[53]
Metabolic flux analysis + ^13^C analysis	Small scale: 73 reactions and 77 metabolites	[54]
Metabolic flux analysis + ^13/14/15^C-labelling	Small scale: 68 reactions and 21 metabolites (19 amino acids)	[55]
Metabolic flux analysis + ^13^C-labelled glucose and glutamine	Small scale: 37 reactions	[56]
Metabolic flux analysis	Small scale: 34 reactions and 30 metabolites	[57]
Metabolic flux analysis in response to varying levels of glutamine supplementation	Small scale: 40 reactions and 37 intracellular, 23 extracellular metabolites	[58]
Metabolic flux analysis to modify medium amino acid composition	Small scale: 23 reactions and 23 metabolites	[59]
Elementary flux mode analysis + kinetic modelling	Small scale: 166 reactions and 29 extracellular, 89 intracellular metabolites	[60]
Flux balance analysis	Genome scale: 1540 reactions and 1302 metabolites	[4]
Flux balance analysis	Genome scale: 1766 genes, 6663 reactions, and 4456 metabolites (in different subcellular compartments)	[61,62,63,64,65]
